# Clinical breast cancer screening uptake and associated factors among reproductive age women in Kenya: further analysis of Kenyan Demographic and Health Survey 2022

**DOI:** 10.1371/journal.pone.0320730

**Published:** 2025-04-01

**Authors:** Habtamu Wagnew Abuhay, Gebrie Getu Alemu, Mekuriaw Nibret Aweke, Tewodros Getaneh Alemu, Berhanu Mengistu, Amare Mesfin

**Affiliations:** 1 Department of Epidemiology and Biostatistics, Institute of Public Health, College of Medicine and Health Sciences, University of Gondar, Gondar, Ethiopia; 2 Department of Human Nutrition, Institute of Public Health, College of Medicine and Health Sciences, University of Gondar, Gondar, Ethiopia; 3 Department of Pediatrics and Child Health Nursing, School of Nursing, College of Medicine and Health Sciences, University of Gondar, Gondar, Ethiopia; Sunu Sante Consulting, SENEGAL

## Abstract

**Introduction:**

Breast cancer is a global public health problem among reproductive-age women, with an estimated 670,000 deaths in 2022. It’s also a pressing health challenge in Sub-Saharan African countries, driven by late-stage diagnoses and limited healthcare access, underscoring the urgent need for early screening and treatment initiatives to combat this growing epidemic. In Kenya, the burden was also significant, and multilevel factors such as individual, household, and community level factors that influence screening uptake are undermined. Furthermore, there were no nationally representative studies. Therefore, this study aimed to assess clinical breast cancer screening uptake (CBCSU) and associated factors among reproductive-age women: further analysis of Kenyan demographic and health survey (KDHS) 2022.

**Methods:**

This study used a weighted nationally representative sample of 16,649 women from the 2022 KDHS. A Multilevel mixed effects binary logistic regression analysis was performed and in the multivariable analysis, variables with a p-value less than 0.05 were considered statistically significant. The strength of the association was evaluated using Adjusted Odds Ratios (AOR) along with their corresponding 95% confidence intervals (CI). STATA version 17 software was to for data management and statistical analysis.

**Results:**

The weighted prevalence of CBCSU in Kenya was 13.91% (95% CI: 13.33, 14.44). Besides, women aged 25 to 34 years (AOR =  1.86, 95% CI: 1.57, 2.21), and 35 to 49 years (AOR =  2.87, 95% CI: 2.40, 3.42) had higher odds of CBCSU. Women with primary education (AOR =  2.14, 95% CI: 1.56, 2.94) and those with secondary or higher education (AOR =  3.09, 95% CI: 2.23, 4.28) had higher odds of CBCSU. In addition, the odds of CBCSU were higher among women with a middle (AOR =  1.41, 95% CI: 1.19, 1.67) and a rich wealth index (AOR =  2.19, 95% CI: 1.84, 2.61). On the other hand, CBCSU was lower among non-contraceptive user women (AOR 0.78, 95% CI: 0.69, 0.87). Furthermore, women in communities with a high proportion of media exposure had higher CBCSU (AOR =  1.17, 95% CI: 1.04, 1.33).

**Conclusion:**

In this study, the prevalence of CBCSU among reproductive-age women in Kenya was found to be low. Besides, factors such as age, educational status, wealth index, family planning utilization, and community media exposure were identified as significant contributors to screening uptake. Therefore, policymakers and stakeholders should design interventions that address factors contributing to low breast cancer screening uptake, particularly targeting women in areas with limited media exposure, to increase the uptake of clinical breast cancer screening.

## Introduction

Breast cancer remains a pressing health challenge for women globally, with an estimated 670,000 deaths in 2022 [1]. In Africa, the burden was also significant, with an estimated 186,598 new cases and, 85,787 deaths reported in 2020 [2]. The high mortality rates in sub-Saharan Africa, driven by late-stage diagnoses and limited healthcare access, underscore the urgent need for enhanced awareness, screening, and treatment initiatives to combat this growing epidemic [3]. Specifically, in Kenya, breast cancer remains a significant public health concern, with an estimated 7,243 new cases and 3,398 associated deaths reported in 2022 [4], these highlight the urgent need for enhanced cancer prevention, and early detection strategies [5].

Clinical breast examinations are recommended at intervals of 1 to 3 years for women aged 25 to 39 and annually for those aged 40 and older [6]. Regular screening is essential for the early detection of breast cancer, which plays a critical role in reducing mortality by enabling prompt intervention and appropriate treatment [7]. Screening practices, however, were often underutilized [8]. The uptake of clinical breast examination (CBE) for breast cancer screening shows substantial variation across regions and income levels [9,10].

In Vietnam, CBCSU was reported at 51% [11], while in the Maldives, it was 41.1% [12]. Across low- and middle-income countries, the self-reported rate of CBCSU was 23.1% [13], This rate was even lower in sub-Saharan Africa, with an overall CBCSU was 14.23% [14]. Furthermore, In Kenya, a sub-Saharan African country, where awareness and access to healthcare services are frequently constrained by socioeconomic, cultural, and systemic barriers as of 2014 only 12% of women had been screened for breast cancer [15].

Studies have shown that early detection through regular clinical breast screening can significantly improve survival rates by facilitating earlier treatment interventions [16,17]. Various sociodemographic factors, including education level, income, residence, and health-seeking behavior, media exposure have been associated with clinical breast cancer screening uptakes [18–20].

Despite the Kenyan government implementing various measures to enhance CBCSU [21], there is limited data on the factors that influence clinical breast cancer screening uptake. Furthermore, multilevel factors such as individual, household, and community-level factors that influence screening uptake are undermined. Additionally, previous studies were limited in population, and outdated, there is a need for further national representative studies.

Therefore, this study aims to assess the prevalence of clinical breast cancer screening uptake and associated factors among reproductive-age women in Kenya. By identifying multilevel factors associated with screening uptake, this analysis seeks to provide evidence to inform targeted public health interventions and policy measures to enhance screening uptake and ultimately reduce breast cancer mortality.

## Methods

### Study design, setting, and period

This study employed a cross-sectional study design based on data from the KDHS conducted in 2022. The KDHS is a nationally representative survey that covers all 47 counties of Kenya and provides comprehensive data on health and demographic indicators, including fertility, mortality, maternal and child health, and access to health services. The survey was conducted by the Kenya National Bureau of Statistics (KNBS) in collaboration with the DHS Program.

### Data source and extraction

The data for this study were obtained from the 2022 KDHS dataset, which is publicly available through the DHS Program website (www.dhsprogram.com). The KDHS uses a stratified two-stage cluster sampling method, ensuring a representative sample of households from both rural and urban areas of Kenya.

Data extraction was performed using Stata version 17, and the dataset was downloaded after receiving permission from the DHS Program. After that, the breast cancer screening uptake and its associated individual and community-level variables were carefully extracted from the Women’s file (IR) aged 15-49 years, in 2022 KDHS.

### Variables of the study

#### Dependent variable.

In this study, the outcome variable focused on clinical breast cancer screening. Clinical breast cancer screening is defined as a physical examination of the breasts conducted by a healthcare provider [22]. It was classified into binary format, where a response of “yes” to the question “breasts examined for cancer by health care provider?” was assigned a value of 1, and a response of ‘no’ was assigned a value of 0.

#### Independent variables.

The independent variables included several sociodemographic and health-related factors based on prior literature and their availability in the KDHS dataset. The variables are grouped into two categories: factors at the individual and community levels. The individual-level variables included maternal age, educational status, marital status, household wealth index, the contraceptive method used, perceived health status, occupation, family size, and distance to health facilities. The place of residence, community media exposure, community educational status, and community wealth index were considered community-level variables.

### Data collection tools and procedures

The KDHS utilized standardized questionnaires developed by the DHS Program, which were pre-tested and validated for use in Kenya. These questionnaires were administered by trained enumerators through face-to-face interviews conducted at selected households. The data collection tools covered various topics, including household demographics, maternal and child health, family planning, and health-seeking behavior.

For this study, the data of interest were collected from the specific sections of the KDHS questionnaire focusing on women’s chronic disease conditions. The enumerators collected data using computer-assisted personal interviewing (CAPI) technology, which minimized errors and ensured data quality.

### Data processing and analysis

Before conducting the analysis, the data were thoroughly cleaned, transformed, and categorized accordingly. Descriptive statistics were then performed and summarized using tables, graphs, and text. Stata version 17 software was used for data extraction and appropriate statistical analysis, with appropriate survey weights applied to account for the complex sampling design and clustering effects. For each variable, descriptive statistics were computed and provided as means and standard deviations for continuous variables and frequencies and percentages for categorical variables.

For inferential analysis, both bi-variable and multivariable logistic regression models were employed to assess the association between independent variables and the outcome of interest (clinical breast cancer screening uptake). Variables with a p-value <  0.2 in the bi-variable analysis were considered for inclusion in the final model of the Multilevel mixed effect binary logistic regression model. Adjusted odds ratio (AOR) with 95% Confidence Interval (CI) was presented, and statistical significance was set at a p-value <  0.05. Results were presented in tables, and figures, and highlighted in the text.

### Multilevel analysis

The DHS data was analyzed using multilevel logistic regression to determine the factors influencing clinical breast cancer screening uptakes. The analysis involved computing the Intra-class Correlation Coefficient (ICC) and Median Odds Ratio (MOR) to measure cluster variation and community-level variability. Additionally, the Proportional Change in Variance (PCV) was calculated to determine how much of the variation in breast cancer screening uptake was explained by the final model.

#### Model building.

In Demographic and Health Survey (DHS) data, individuals are nested within clusters, meaning that individuals within the same cluster share more similar characteristics compared to those in different clusters. This clustering leads to a violation of the standard regression assumptions, particularly the independence of observations and the homogeneity of variance.

To address this issue, a multilevel mixed-effects binary logistic regression model was employed in this study to estimate the association between individual-level and community-level variables and the likelihood of clinical breast cancer screening uptake. Four models were initially fitted:

• Model I (Null model) included no explanatory variables and was used to assess the variability in clinical breast cancer screening uptake across clusters.
• Model II incorporated only individual-level variables.
• Model III included only community-level variables.
• Model IV integrated both individual and community-level factors simultaneously.

Given the hierarchical structure of the data, model comparisons were made using deviance and log-likelihood ratio tests. The comparison determined that Model IV, which included both individual and community-level variables, provided the best fit, as evidenced by the highest likelihood and lowest deviance values.

#### Parameter estimation method.

In this study, the fixed effect, representing the measure of association, was used to estimate the association between the likelihood of clinical breast cancer screening uptake and various explanatory at both individual and community level factors. Factors with a p-value ≤  0.20 were selected as candidates for inclusion in the final model. Both the Crude odds ratio (COR) and the AOR were assessed and the final results were presented as AOR. The associations between clinical breast cancer screening uptake and independent variables were assessed and the strength of these associations was reported using AORs with a 95% CIs. A significance level of p <  0.05 was used to determine statistical significance.


$$\log(\frac{\text{πij}}{1-\text{πij}})=\text{yij }=\text{βo}+\text{β}1\text{xij}+\text{uj}$$

Where Yij represents the clinical breast cancer screening uptake for the i-th woman in the j-th cluster or community.; Xij are individual and community level variables for the i-th individual in the j-th group. β0 is the intercept, indicating the effect on screening uptake in the absence of explanatory variables. β1 are the fixed coefficients, indicating the unit change in the explanatory variables xij and the corresponding effect on the probability of clinical breast cancer screening uptake. uj represents the random effect for the j-th community [23].

For this case, ICC, PCV, and MOR were computed for the sake of identifying variability among clusters (measure of variation).

The ICC which represents the proportion of the between-cluster variation in the total variation (the between- plus the Within-Cluster variation) of the chances of breast cancer screening uptake, should be above 10%. It is calculated as;


$$ICC=\frac{\text{VA}}{\text{VA}+3.29}\text{*}100\text{\% }$$

Where; VA, mean cluster level variance [24].

The MOR is defined as the median value of the odds ratio between the area at the highest risk and the area at the lowest risk when randomly picking out two clusters.


$$MOR=\text{exp}.\left[\surd \left(2\times \text{VA}\right)\times 0.6745\right],\text{or}MOR=e^{0.95\surd VA}$$

where; VA is the area level variance [25].

The PCV reveals the variation in the prevalence of clinical breast cancer screening uptake among reproductive-aged women explained by factors. The PCV is calculated as;

$PCV=\left(\frac{Vnull-VA}{Vnull}\right)*100$ Where; Vnull, means variance of the initial (null) model, and VA, is the mean variance of the model with explanatory variables [25,26].

### Ethical considerations

This study was based on secondary data analysis of Demographic and Health Surveys, therefore permission for access to the data was approved from Measure DHS (ICF International) by registering and stating the purposes of the study. The data used in this study are freely available, aggregated secondary data that didn’t contain any personal identifiers that can be linked to the study participants (http://www.dhsprogram.com). The data were used exclusively for the registered research topic and were not shared with other parties. Complete information about the ethical issue can be found in the KDHS 2022 report.

## Results

### Socio-demographic characteristics of study participants

A total weighted sample of 16,649 women aged 15–49 was included in this study, among them, 3,102 (19%) belonged to the 15–19 age category. The mean age of the participants was 29 years, with a standard deviation of ± 9.54 years. More than half of the study participants 9285 (56.0%) were married, and one-third of study participants 5533 (33.0%) were from poor wealth index families. Approximately half of the study participants, 8,838 (53.5%), did not use contraceptive methods, and four-fifths of 12,995(78.0%) the women reported poor self-rated health status (**Table 1).**

**Table 1:** Breast cancer screening uptake by socio-demographic characteristics in Kenya, 2022 KDHS.

Variables	Category	Weighted frequency (%)
Age	15 - 19	3102(19)
	20 - 24	3053(18)
	25 – 29	2903(17)
	30 – 34	2359(14)
	35 – 39	2283(14)
	40 – 45	1609(10)
	45 – 49	1339(8)
Educational level	Not-educated	916(6)
	Primary	6078(37)
	Secondary	6460(39)
	Higher	3196(19)
Marital status	Single	5319(32)
	Married	9285(56)
	Widowed	450(3)
	Divorced	1595(10)
Occupation	Not employed	7805(47)
	Employed	8829(53)
Wealth index	Poor	5533(33)
	Middle	3076(18)
	Rich	8040(48)
Contraceptive method	Using modern method	7051(42)
	Using traditional method	761(5)
	Does not intend to use	8838(53)
Self-reported health status	Bad	12995(78)
	Moderate	3229(19)
	Good	426(3)
Family size	<= 4	14576(88)
	5 - 8	1931(12)
	>= 9	142(1)
Distance to the health facility	Big problem	3944(24)
	Not- a big problem	12705(76)
Community level variables		
Residence	Urban	6835(41)
	Rural	9814(59)
Proportion of women with media exposure	Low	6924(42)
	High	9725(58)
Proportion of women with primary and higher education	Low	13105(79)
	High	3544(21)
Proportion of women with a poor wealth index	Low	7445(45)
	High	9204(55)

Prevalence of clinical breast cancer screening uptake among Kenyan women.

**Fig 1:**
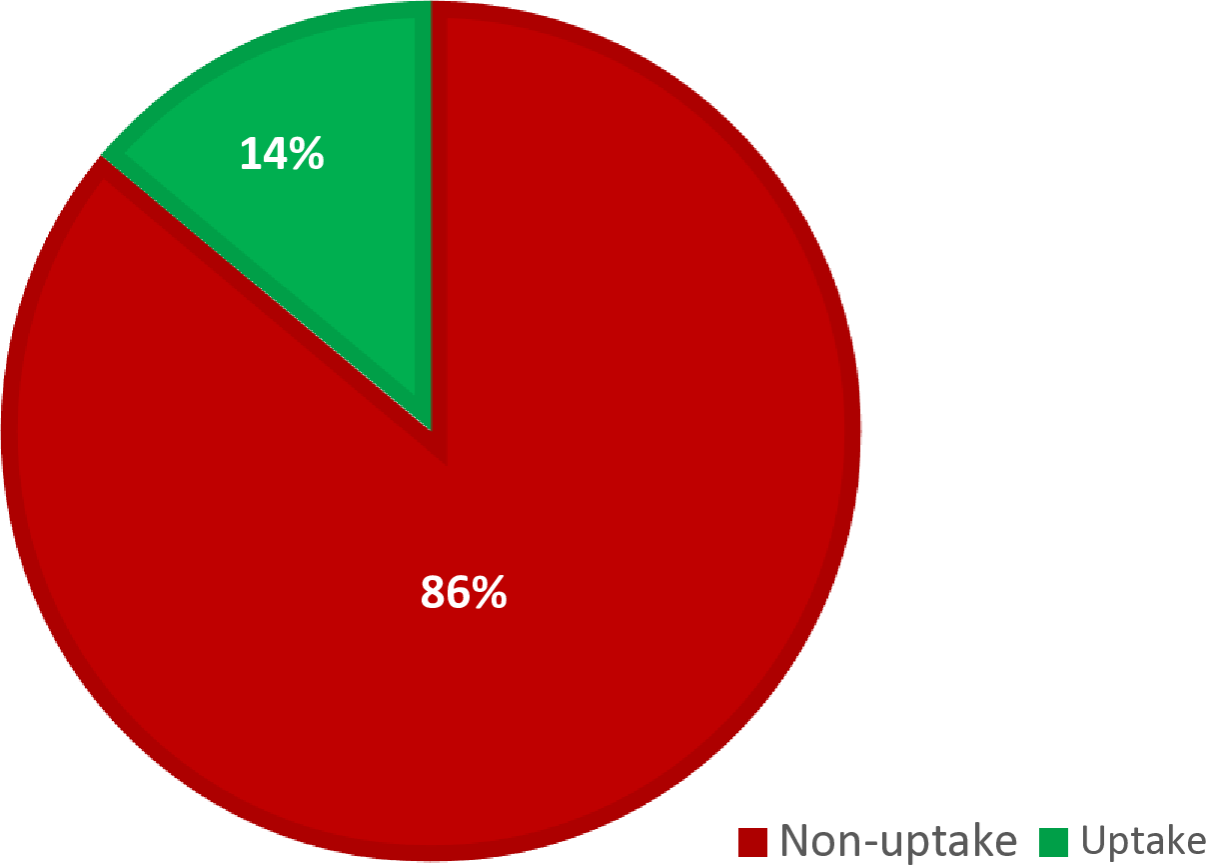
Prevalence of clinical breast cancer screening uptake among reproductive-age women in Kenya, 2022 KDHS.

In this study, the weighted prevalence of clinical breast cancer screening uptake among reproductive-age women in Kenya was 13.91% (95% CI: 13.33% - 14.44%) (**Fig 1).**

Multi-level analysis of factors associated with breast cancer screening uptake.

### Random effect and model comparison

The intra-cluster correlation (ICC) in the null model indicated a significant clustering effect, with an ICC value of 17.4%, exceeding the threshold of 10% [27]. The Median Odds Ratio (MOR) value of 2.209 indicates significant variation in breast cancer screening uptake across clusters. This indicates that if two women are randomly selected from different clusters, the woman from a cluster with a higher prevalence of breast cancer screening has 2.209 times higher odds of utilizing screening compared to a woman from a cluster with a lower prevalence.

The Proportional Change in Variance (PCV) value from the final model indicates that community and individual-level factors explain about 60.7% of the variation in breast cancer screening uptake. This means that after accounting for these factors, 60.7% of the variability in the likelihood of using breast cancer screening across different clusters has been explained.

Furthermore, we fitted and compared the models using the deviance and likelihood tests, determining that Model 4 was the better-fitted model with the highest likelihood and lowest deviation values (**Table 2).**

**Table 2:** Random effect analysis result and model fit statistics for multi-level models.

Parameters	Model 1 (Null)	Model 2	Model 3	Model 4
Variance	0.696	0.271	0.400	0.273
ICC	0.174	0.076	0.108	0.076
MOR	2.209	1.639	1.823	1.642
PCV(%)	Reff	61.1%	42.5%	60.7%
Model fitness				
Likelihood	-5792.381	-5138.463	-5633.052	-5128.494
Deviance	11,594.763	10,276.926	11,266.104	10,256.988

ICC: Intra-class Correlation; MOR: Median Odd Ratio; PCV: Proportional Change *in* Variance

### Fixed effect analysis results

The final model (model-4) demonstrates the incorporation of individual and community-level predictors into the multi-level analysis. Women’s age, educational status, wealth status, family planning methods, and media exposure were identified as significant factors associated with breast cancer screening uptake.

The odds of breast cancer screening uptake were 1.86 times higher among women aged 25 to 34 years (AOR =  1.86, 95% CI: 1.57, 2.21) and 2.87 times higher among women aged 35 to 49 years (AOR =  2.87, 95% CI: 2.40, 3.42), compared to women aged 15 to 24 years.

Women with primary education and those with secondary or higher education had 2.14 times (AOR =  2.14, 95% CI: 1.56, 2.94) and 3.09 times (AOR =  3.09, 95% CI: 2.23, 4.28) higher odds of breast cancer screening uptake, respectively, compared to not-educated women. The odds of clinical breast cancer screening uptake were 1.41 times higher among women with a middle wealth index (AOR =  1.41, 95% CI: 1.19, 1.67) and 2.19 times higher among women with a rich wealth index (AOR =  2.19, 95% CI: 1.84, 2.61), compared to those with a poor wealth index.

The odds of clinical breast cancer screening uptake were 22% lower among non-contraceptive user women (AOR 0.78, 95% CI: 0.69, 0.87) than modern contraceptive user women. Furthermore, regarding community-level factors, women in communities with a high proportion of media exposure had 1.17 times higher odds of clinical breast cancer screening (AOR =  1.17, 95% CI: 1.04, 1.33) than women in communities with low media exposure. (**Table 3).**

**Table 3:** Multilevel analysis for factors associated with clinical breast cancer screening uptakes among women in Kenya, 2022 KDHS.

Variable	Category	Model I Null model	Model II	Model III	Model VI
			AOR [95% CI]	AOR [95% CI]	AOR [95% CI]
Age	15 – 24	–	1	–	1
	25 – 34	–	1.88[1.58, 2.23]	–	1.86[1.57, 2.21]^*^
	35 – 49	–	2.91[2.44, 3.48]	–	2.87[2.40, 3.42]^*^
Educational Status	Not educated	–	1	–	1
	Primary	–	2.62[1.95, 3.52]	**–**	2.14[1.56, 2.94]^*^
	Secondary and higher	–	3.85[2.85, 5.20]	–	3.09[2.24, 4.28]^*^
Marital status	Married	–	1	–	1
	Single		0.65[0.54, 0.77]	–	0.93[0.53, 1.75]
	Divorced	–	1.12[0.95, 1.32]	–	0.97[0.72, 1.30]
	Widowed	–	0.98[0.73, 1.32]	–	1.12[0.94, 1.31]
Occupation	Un-Employed		1		1
	Employed		1.77[0.56, 2.01]		1.85[0.85, 2.48]
Wealth index	poor	–	1	–	1
	Middle	–	1.46[1.23, 1.72]	–	1.41[1.19, 1.67]^*^
	Rich	–	2.37[2.04, 2.76]	–	2.19[1.84, 2.61]^*^
Contraceptive Method	Modern- Method	–	1	–	1
	No-users	–	0.76[0.68, 0.86]	–	0.78[0.69, 0.87]^*^
	Traditional- Method	–	1.09[0.86, 1.37]	–	1.09[0.86, 1.37]
Self-reported health status	Good	–	1	–	1
	Bad	–	0.84[0.61, 1.15]	–	0.83[0.61, 1.14]
	Moderate		0.97[0.71, 1.35]		0.96[0.69, 1.34]
Distance to the health facility	Big problem	–	1	–	1
	Not a big problem	–	0.97[0.85, 1.11]	–	0.95[0.83, 1.09]
Community level variable					
Residence	Urban	–	–	1	1
	Rural	–	–	0.61[0.53, 0.69]	0.94[0.81, 1.091
Proportion of women with primary and higher education	Low	–	–	1	1
	High	–	–	0.43[0.36, 0.50]	0.98[0.76, 1.12]
Proportion of women with media exposure	Low	–	–	1	1
	High	–	–	1.37[1.20, 1.56]	1.17[1.04, 1.33]^*^

1: Reference, AOR: Adjusted odds ratio, CI: Confidence interval,

* p-value <  0.05

## Discussions

Despite the WHO recommendation for women to undergo at least an annual clinical breast examination, our findings indicate that non-uptake of breast cancer screening remains a significant public health concern. The low screening rates not only have severe consequences for women’s health but also place a substantial burden on their families and the national healthcare system. This study investigated the uptake of clinical breast cancer screening and its associated factors among reproductive-age women in Kenya. The findings provide substantial insights into screening behaviors and highlight several factors that influence the uptake of breast cancer screening. These findings underscore the need for focused interventions to promote screening uptake and early detection, particularly in populations with low involvement in preventive health measures.

In this study, the weighted prevalence of CBCSU among reproductive-age women in Kenya was 13.91% (95% CI: 13.33, 14.44%). This finding is higher than previous studies conducted in underdeveloped nations[28], and Lesotho were (11.4%) and 9.7% [18]. The difference in clinical breast examination rates could be due to the timing of the studies. Over time, increased awareness from health campaigns, and enhanced healthcare access, have likely contributed to higher rates in this study. But, this study result is lower than a study conducted in Nigeria 16.5% [29], Thailand 40.1% [30], and Vietnam 51% [11]. This might be due to differences in limited healthcare infrastructure, socioeconomic barriers, cultural stigma, and a lack of emphasis on preventive care have kept CBE rates lower in Kenya compared to developed countries, where preventive healthcare is more accessible and routinely encouraged.

Additionally, this study revealed several key factors associated with CBCSU. Both individual and community-level factors, such as women’s age, educational status, wealth status, family planning strategies, and community media exposure, were found to be significant factors associated with CBCSU.

In this study, women aged 25–34 and 35–49 years demonstrated significantly higher odds of CBCSU compared to those aged 15–24 years. This finding aligns with a study conducted in Tanzania, which observed a higher rate of breast cancer screening among women aged 35–49 years [31]. Similarly, a study in Spain revealed that older age was associated with increased breast cancer screening uptake [32]. This could be due to the fact that older women may have increased awareness and understanding of the risks associated with breast cancer, leading them to seek screening more proactively [33]. Additionally, family responsibilities might drive older women to prioritize their health, contributing to higher screening uptake in these age groups. Furthermore, the higher health-seeking behavior of older women may contribute to the increased uptakes of clinical breast cancer screening uptake [34].

Compared to women with no formal education, women with primary or higher education had higher CBCSU. This finding is supported by previous studies in Ghana [35] and Bangladesh [36], which also found that women with higher levels of education were more likely to receive screening services. This may be because educated women are generally more informed about the benefits of early detection, understand the importance of preventive healthcare, and may feel more empowered to seek out these services [37]. Additionally, education improves women’s access to health information and empowers them to make informed health decisions, leading to higher breast cancer screening uptake [38].

This study found that women from a middle and rich wealth index family had higher odds of CBCSU, compared to those with a poor wealth index. These results were consistent with findings from Botswana [39], which showed that the odds of breast cancer screening were low among women in the poorest wealth quintiles. Also, other studies in Ghana revealed that women within the richest wealth index had increased odds of screening for BC compared to those in the poorest wealth index [40].

Possibly wealthier women are more likely to have health insurance or financial resources that facilitate access to and utilization of healthcare services [41]. In contrast, women from poorer households may face barriers such as cost, lack of transportation, or time constraints, leading to lower screening uptake [42]. Addressing these barriers can help achieve more equitable breast cancer screening, improving early detection and outcomes, especially for women from disadvantaged backgrounds.

The results of this study demonstrate that women who do not use contraceptives had lower CBCSU compared to women who use modern contraceptives. This finding is consistent with previous research conducted in sub-Saharan Africa, which identified a positive association between contraceptive use and an increased likelihood of undergoing breast cancer screening [43]. This could be because women who use contraceptive services are more engaged with healthcare systems. As a result, they may be more likely to participate in other preventive health measures, such as breast cancer screening.

Furthermore, regarding community-level factors, our study found that women residing in communities with a higher proportion of media exposure exhibited significantly greater uptake of CBCSU than those in communities with limited media exposure. This result aligns with a study from Lesotho, which demonstrated that women exposed to media were more likely to participate in CBE than their counterparts with minimal media exposure [18]. This finding underscores the crucial role of media in disseminating health information and promoting positive health behaviors, likely increasing awareness and uptake of breast cancer screening.

Strength and limitation: The key strength of this study was that it employed a nationally representative sample and the rigorous methodology, including an appropriate statistical analysis that took into account the data’s hierarchical structure, and the practical recommendations that were provided for policymakers and public health stakeholders However, this study had limitations in that Secondary data is used which can restrict the depth of analysis and limit the exploration of variables not included in the original survey. Moreover, other important factors like cultural beliefs and attitudes towards screening have not been explored in detail.

## Conclusion and recommendation

In this study, the prevalence of clinical breast cancer screening uptake among reproductive-age women in Kenya was found to be low. Besides, factors such as age, educational status, wealth index, family planning utilization, and community media exposure were identified as significant contributors to screening uptake.

These findings highlight the need for evidence-based government strategies, including expanding screening programs and targeted awareness campaigns. Addressing financial barriers is crucial to ensuring equitable access, particularly among vulnerable populations. Therefore, Policymakers should utilize these insights to design more effective programs that improve awareness and access for high-risk groups. Furthermore, future research should focus on evaluating cultural barriers and examining the impact of policies on breast cancer screening uptake were strongly recommended.

## Abbreviations

ANC: Antenatal care
CBCSU: Clinical breast cancer screening uptake
CSA: Central statistical agency
EAs: Enumeration areas
KDHS: Kenyan demographic and health survey
ICC: Intra-class Correlation
ICF: International Classification of Functioning
LLR: Log-likelihood ratio
MOR: Median Odd Ratio
PCV: Proportional Change in Variance
SD: Standard Deviation
UNICEF: United Nation International Children’s Fund
VIF: Variance Inflation Factor
WHO: World Health Organization
